# Microbial and Chemical Stability of Unpreserved Atropine Sulfate 0.01% *w*/*w* Eye Drops—A Pilot Study on the Impact of Dispenser Type and Storage Temperature over 12 Weeks of Daily Use After Compounding

**DOI:** 10.3390/life15111646

**Published:** 2025-10-22

**Authors:** Victoria Klang, Stefan Brenner, Johanna Grabner, Philip Unzeitig, My Vanessa Nguyen Hoang, Maria Lummerstorfer, Roman Pichler, Katja Steiner, Richard D. Harvey

**Affiliations:** 1 Department of Pharmaceutical Sciences, University of Vienna, Josef-Holaubek-Platz 2, 1090 Vienna, Austria; 11727507@unet.univie.ac.at (J.G.); a01427357@unet.univie.ac.at (P.U.); my.nguyen@univie.ac.at (M.V.N.H.); maria.lummerstorfer@univie.ac.at (M.L.); katja.steiner@univie.ac.at (K.S.); richard.harvey@univie.ac.at (R.D.H.); 2 Laboratory of the Austrian Pharmacy Chamber Vienna, Spitalgasse 31, 1090 Vienna, Austria; stefan.brenner@apothekerkammer.at (S.B.); roman.pichler@apothekerkammer.at (R.P.); 3 Vienna Doctoral School of Pharmaceutical, Nutritional and Sport Sciences, University of Vienna, Josef-Holaubek-Platz 2, 1090 Vienna, Austria

**Keywords:** progressive myopia, atropine sulfate, homatropine hydrobromide, in-use stability, preservative-free dispenser, pharmaceutical compounding, contamination stress test

## Abstract

Progressive myopia in children is a highly prevalent condition in societies worldwide and is often treated with compounded low-dose atropine sulfate (AS) eye drops without preserving agents to avoid irritation/sensitisation. Surprisingly, there is a lack of data regarding the in-use stability of contamination-free LDPE dispenser units (CFDs) for this compounded multidose product, which causes uncertainty among prescribers and patients in Europe. Thus, our aim was to compare the effect of different dispenser types on the chemical and microbial stability of unpreserved AS eye drops (0.01% *w*/*w*). A dripping simulation was performed to obtain information on microbial stability over 4 weeks through plating and separately over 12 weeks through direct inoculation, HPLC and pH analysis. For CFDs, no contamination was found after 4, 8 or 12 weeks of use when stored at 23 or 4 °C as opposed to the control. AS content remained within 0.01 ± 0.0002% *w*/*w* after 12 weeks, with higher chemical stability at 4 °C despite decreasing pH. A stress test confirmed the validity of the CFD system. In conclusion, using CFDs and refrigerated storage was found to be safe for compounded unpreserved AS eye drops over 12 weeks of use.

## 1. Introduction

In paediatric healthcare, there is an increasing demand for specific pharmaceutical products to treat acute and chronic conditions. Frequently, however, no marketed product is available, such as in the case of atropine sulfate eye drops. Atropine sulfate (AS) has been used for diagnostic purposes alongside other mydriatic drugs for decades. High-dose treatment with the muscarinic receptor antagonist AS (0.5–1.0% *w*/*w*) has been explored [1,2], is well documented and currently among the most effective and most researched pharmacological interventions in clinical practice for slowing the advancement of refractive error and ocular axial growth [3,4,5,6]. However, it is associated with side effects such as allergic reactions, glare, near visual loss and rebound axial growth after cessation [4,7,8]. In recent years, the use of low-dose AS eye drops (0.01% *w*/*w*) against progressive myopia in children has been introduced to the Western medical market from East and Southeast Asia, where a number of short-sighted school children in urban environments are nearing 50% [9,10,11,12,13]. Several meta-analyses indicate positive clinical outcomes for this low-dose strategy [14,15,16], which has the advantage of reduced risk of side effects and rebound effect [4].

Since no ophthalmological pharmaceutical products containing AS are approved by authorities and registered on the broader Asian, US or European markets, low-dose AS eye drops are being compounded in daily practice either in hospital or community pharmacies, of which over 1400 exist in Austria alone. The common practice is aseptic production by diluting marketed or compounded AS stock solutions (e.g., solutions for injection, 0.1 or 0.5% *w*/*w* [11]) using sterile filter units. Although the shelf life of unopened sterile eye drops produced under aseptic conditions has been published in the Austrian pharmacopoeia as well as in international studies [13], very little data exist on the chemical and microbial stability of AS eye drops under daily use conditions.

In the academic literature, only test scenarios with very specific conditions have been explored so far (PET bottles, 15 mL, ethylene oxide sterilisation [11]), which do not comply with regulatory standards in Austria and Germany. Knowledge about in-use shelf life, however, is highly relevant for consumer safety and adherence. Indeed, contradictory information concerning in-use shelf life has been circulating among parents and practitioners, hindering efficient and safe long-term treatment.

Therefore, it was the aim of this study to establish the in-use chemical and microbial storage stability of compounded AS (0.01%) eye drops in 10 mL packing units under conditions mimicking daily use over a 12-week period. In addition, the impact of the dispenser system (contamination-free dispenser unit vs. control amber glass dispenser unit with BIIR cone), as well as the impact of storage temperature (23 °C vs. 4 °C), was determined to clarify optimal production conditions for practitioners and pharmacists, as well as optimal storage conditions for patients at home.

The obvious clinical need, as well as the scant data available, render the presented stability study on the in-use shelf life of compounded AS (0.01% *w*/*w*) eye drops an important asset to current clinical practice in Austria, but also internationally. New aspects of this study include the validation of products from small-scale production, as commonly performed in Austrian pharmacies, and assessing the impact of different dispensing units on in-use storage stability. To our knowledge, no data on the chemical stability of aqueous AS eye drops in contamination-free LDPE dispensers under prolonged simulated use conditions exist in the literature; we therefore aim to close this knowledge gap to improve patient care and adherence in regional and international compounding practice.

## 2. Materials and Methods

### 2.1. Materials

Atropine sulfate monohydrate (USP, CAS-Nr. 5908-99-6) was obtained from Gatt-Koller GmbH (Absam, Austria). Purified water for injection was obtained from B.Braun (Melsungen, Germany). Sterile pyrogen-free isotonic saline was obtained from Fresenius Kabi AG (Bad Homburg, Germany).

All microbiological culture media and supplements were obtained from Carl Roth GmbH + Co. KG (Karlsruhe, Germany). For bacterial cultures, agar consisting of casein peptone, glucose monohydrate and agar (CASO, Ph. Eur./USP, ISO 9308 [17], ISO 11133 [18], ISO 11930 [19], ISO 18415 [20], ISO 18416 [21], ISO 21149 [22], ISO 21150 [23], ISO 22717 [24], ISO 22718 [25]) and Lennox LB-Agar consisting of tryptone, malt extract, sodium chloride and agar were used. For fungal cultures, Sabouraud 4% dextrose agar consisting of peptone from casein, peptone from soja, sodium chloride and agar (SAB, Ph. Eur./USP, ISO 11133 [18], ISO 16212 [26]) was used. For the sterility tests according to Ph.Eur. (2.6.1.), CASO bouillon (CASO-B, Ph. Eur./USP, ISO 10273 [27], ISO 11133 [18], ISO 21871 [28]) consisting of peptone from casein, peptone from soja, potassium hydrogen phosphate, sodium chloride and glucose monohydrate was used. All other reagents were pharmaceutical grade and used as obtained. *Escherichia coli* (*E. coli*) DH5α was purchased from Thermo Fisher Scientific (Waltham, MA, USA). The agar plating was performed by using Petri dishes without ventilation caps from Sarstedt AG & Co. KG (Nümbrecht, Germany).

Novelia contamination-free dispensing units (CFD) were kindly provided by Elpack GmbH (Lieboch, Austria), consisting of a sterile LDPE bottle (11 mL) with HDPE/silicone dispensing unit and HDPE cap. Sterile brown glass (BG) bottles (10 mL, class I) and sterile dripping cones made from BIIR (bromobutyl rubber, a brominated copolymer of isobutylene and isoprene) were obtained from Herba Chemosan Apotheker AG (Vienna, Austria).

### 2.2. Production of Eye Drop Solutions

Physico-chemical and microbial stability were assessed after production from a 1% *w*/*w* stock solution of AS using 0.9% NaCl to a final concentration of 0.01% *w*/*w*, followed by sterile filtration through cellulose acetate filters (pore size 0.22 µm) and aseptic filling of 10 mL into sterile LDPE bottles (11 mL) or sterile brown glass bottles (10 mL, class I).

AS eye drops were produced in batches as follows: AS stock solutions using water for injection were produced at 1% *w*/*w* using magnetic stirring for 5 min at 23 °C. Stock solutions were diluted to 0.01% *w*/*w* using isotonic pyrogen-free saline. The resulting transparent AS eye drop solution (40.0 g of 0.01% AS) was stirred for 5 min at 23 °C and then filtered into sterile eye drop dispensers using a laminar air flow (LAF) cabinet (MSC Advantage 1.5, Thermo Electron LED GmbH, Langenselbold, Germany), a sterile syringe (Omnifix Luer Lock Solo 20 mL, B.Braun, Melsungen, Germany) and cellulose acetate filter unit (ReliaPrep Syringe Filter single-use non-pyrogenic, Ahlstrom, Bärenstein, Germany). From each batch, four dispenser units filled with 10.0 g AS eye drops—2 CFD, 2 BG—were obtained through aseptic filtration under the LAF.

### 2.3. Storage and Study Design of Daily Use Simulation

To investigate the impact of storage temperature on chemical and microbial stability, the different dispenser units were either stored at room temperature (23 ± 1 °C) or refrigerated (4 ± 1 °C), both under protection from light.

To simulate daily patient use, a sampling routine was performed at the same time point daily from Monday to Friday by disposing of two individual drops from each dispenser. General hygiene was maintained, but the procedure was performed under normal laboratory conditions (23 °C, no aseptic environment, no disposable gloves). Eye drop samples stored at 4 °C were acclimatised to room temperature (23 °C) for 10 min before use, before being returned to the refrigerator for storage at 4 °C. Preliminary experiments were conducted to determine droplet mass per dose of the different dispenser types.

### 2.4. Microbial Stability Monitoring

Microbial stability of the 0.01% *w*/*w* AS eye drop formulations was determined during in-use simulation to investigate differences between the two dispenser types (CFD, BG) and storage temperatures (23 °C and 4 °C). To this end, two methods were employed.

**Figure 1:**
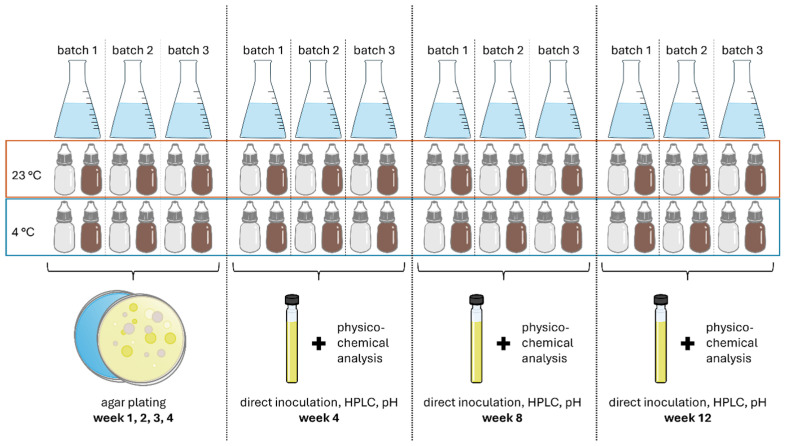
Illustration of study design for stability monitoring of eye drop solutions in brown glass (BG) dispensers or contamination-free LDPE Novelia^®^ dispenser units (CFD).

First, three individual batches of AS eye drops were analysed during use over 28 days through regular plating (day 7, 14, 21, 28). Then, long-term monitoring was performed for three individual batches of AS eye drops over, respectively, 4, 8 and 12 weeks of use (Figure 1).

For these tests, different culture media were used: CASO and Lennox LB agar were used as general bacterial culture media. Sabouraud dextrose agar, containing dextrose as energy source, peptone as nitrogen source and offering low pH, is recommended for the selective cultivation of yeasts, moulds and aciduric bacteria; it is particularly useful for cultivation of fungi associated with skin infections. CASO-B nutrient broth was used to promote growth of a wide range of non-fastidious organisms. The studies were carried out under aerobic conditions to reflect real in-use circumstances in clinical practice [29].

#### 2.4.1. Agar Plating and CFU Counting

Plating was conducted in weekly intervals on days 7, 14, 21 and 28 over 4 weeks during daily dripping simulation by using the pour plate method [30], which is recommended by the Food and Drug Administration (FDA).

To this end, 0.9 mL of fluid eye drop sample dripped from the dispenser was pipetted into agar plates and mixed with 20 mL of sterile autoclaved medium (Hiclave HV-58L, HMC Europe, Tuessling, Germany). The inoculated media were allowed to dry prior to incubation. CASO and LB agar were used for general bacterial growth and incubated at 30 ± 1 °C for 3 days (drying cabinet UE500, Memmert GmbH, Büchenbach, Germany). Sabouraud agar was used as nutrient medium for specific fungal detection and incubated at 21± 1 °C for 6 days (drying cabinet ED115, Binder GmbH, Tuttlingen, Germany). After this time span, plates were inspected visually for growth of bacteria or fungi by counting the number of colony-forming units (CFUs). To test the agar functionality, positive controls were performed by using tap water, and negative controls were performed by using sterile water for injection.

#### 2.4.2. Direct Inoculation (Ph.Eur. 2.6.1)

Eye drops were checked for microbial contamination after daily use for 28, 56 and 84 days (weeks 4, 8 and 12). Analysis after 4, 8 and 12 weeks was, respectively, conducted in triplicate on separate batches (Figure 1) to ensure sufficient residual volume for analysis (pH, HPLC). The method used was harmonised according to the *European Pharmacopoeia* methodology 2.6.1. for sterility testing using direct inoculation.

Nutrient media were prepared by autoclaving 10.0 mL of fluid CASO broth directly in separate test tubes (Hiclave HV-58L, HMC Europe, Germany). For inoculation, 1.0 mL of fluid eye drop sample dripped from the dispenser was pipetted into the test tubes using aseptic preparation in the LAF. As a reference, nutrient broth samples were inoculated with purified water for injection (negative control) and tap water (positive control). The test tubes were incubated at 30 ± 1 °C for 14 days. Visual inspection for signs of microbial contamination was performed regularly under standardised conditions (days 1, 3, 7, 14). Transparency and colour were confirmed; the presence/absence of visible particles and turbidity or haziness were documented.

#### 2.4.3. Tip Seal Integrity Test (TSIT)

To additionally check the risk of microbial contamination with the different dispenser types, a microbial tip seal challenge test was conducted (tip seal integrity test or TSIT 2.0. [31] after contamination challenge strategies suggested by Bagel et al. [32]). Before the test, sterile fluid medium CASO-B (30 g/L purified water) was prepared, cooled to room temperature, and 10 mL of medium was filled into CFD and BG dispenser units under aseptic conditions in the LAF. *E. coli* DH5α were cultivated in LB medium at 37 °C with constant shaking and diluted regularly with sterile medium to ensure log-phase growth.

**Figure 2:**
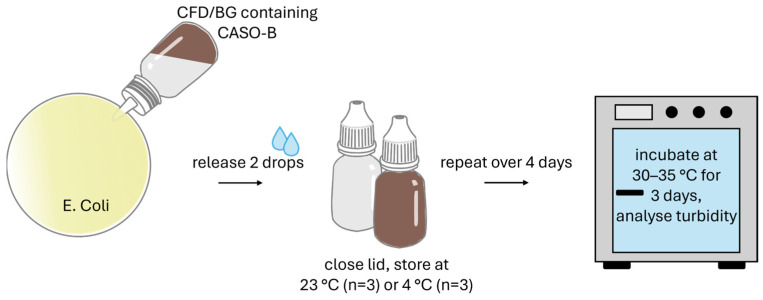
Illustration of tip seal integrity testing for the two dispenser types (BG = brown glass; CFD = contamination-free dispenser).

The contamination challenge (Figure 2) was performed over 3 days with 3× contamination simulations per day, separated by 3 h intervals. During this time, samples were stored at either 23 ± 1 °C or 4 ± 1 °C under protection from light. For each contamination, the dispenser unit was opened, the tip was dipped into 40 mL of *E. coli* suspension (5 × 10^6^ CFU/mL) in a Petri dish, and two droplets were applied into the dish before the dispenser was re-closed. On day 4, this procedure was conducted once more to achieve a 10× contamination regime before the incubation phase was started (3 days of incubation at 30–35 °C). Also, CFD and BG control dispenser units were filled with 10 mL of pure sterilised CASO-B medium and were stored and incubated in the same way as the test samples, but without being opened (negative controls). As positive control, dispenser units were filled with CASO-B and 50 µL *E. coli* suspension (5 × 10^6^ CFU/mL).

After the incubation phase, optical evaluation of the sample turbidity was conducted.

### 2.5. Chemical Stability Monitoring Through HPLC: Validation and Analysis

AS content in the eye drop solutions was monitored during storage after, respectively, 4, 8 and 12 weeks of in-use simulation using high-performance liquid chromatography (HPLC). For an adapted protocol following the directives of the USP monograph, atropine sulfate ophthalmic solution was used. Samples were analysed using a Shimadzu HPLC instrument with diode array detection (2 × SHIMADZU degasing unit DGU-20A5R, 1 × SHIMADZU degasing unit DGU-20A3R, SHIMADZU LC-30AD pump A, SHIMADZU LC-30AD pump B, SHIMADZU communication bus module, SHIMADZU SIL-30AC autosampler, SHIMADZU RF-20A XS fluorescence detector, SHIMADZU SPD-M20A diode array detector, SHIMADZU CTO-30A column oven, Shimadzu Ges.m.b.H., Korneuburg, Austria). A cyano-phase column in reversed-phase mode (Luna CN, 100 mm × 4.6 mm × 3 µm, Phenomenex Ltd., Torrance, CA, USA) was used as the stationary phase. The mobile phase consisted of methanol/sodium acetate buffer pH 4.5 (11:89 *v*/*v*; buffer composition: 6.8 g sodium acetate trihydrate, 3.5 mL triethyl amine, 6.6. mL glacial acetic acid per 1 L of distilled water). After degassing, samples were injected at a flow rate of 1.2 mL/min at 40 °C, with the injection volume set at 50 µL. Chromatograms were analysed at 225 ± 8 nm. Peak areas (AUC) were obtained using the Chromeleon 7 Chromatography Data System (Thermo Scientific Chromeleon Version 7.2.10 ES). Samples were analysed for atropine sulfate content and content of known degradation products (tropic acid (3-hydroxy-2-phenylpropanoic acid), atropic acid (2-phenylacrylic acid) and apoatropine ((1R,3r,5S)-8-methyl-8-azabicyclo(3.2.1)octan-3-yl-2-phenylacrylate)). Drug quantification was performed using reference solutions within a calibration range of 40–120 µg/mL and an R^2^ of 0.999.

### 2.6. Osmolality and pH Assessment

Osmolality of the 0.01% *w*/*w* AS eye drop solutions was determined in triplicate after production using an osmometer (K-7400S Semi-Micro Osmometer, Knauer, Berlin, Germany). For stability monitoring, pH was measured during production and after the in-use simulation after 4, 8 and 12 weeks (SevenCompact™ pH meter, Mettler-Toledo GmbH, Columbus, Ohio, USA).

### 2.7. Acceptability Criteria: In-Use Shelf Life After Opening

The in-use stability of the aqueous AS (0.01% *w*/*w*) eye drops was determined by regular assessment of the following endpoints during daily use conditions: optical transparency through visual inspection, stability of pH and AS content, as well as analysis of degradation products and analysis of microbial contamination using agar plating and direct inoculation.

This study was conducted following regulatory guidelines and published methods of the *European Pharmacopoeia*. Chemical stability of AS was deemed acceptable within boundaries of 90 and 110% of initial drug content [13,29]. Eye drop samples were required to be particle-free, optically transparent and colourless. The observed pH was likewise deemed acceptable within 90 and 110% of the initial value. Microbial stability and safety of use were deemed acceptable if the absence of bacteria and fungi was confirmed using the methods described.

### 2.8. Statistics

Results are expressed as statistical mean ± standard deviation where applicable. Data analysis was performed using GraphPad Prism 3.0 software (GraphPad Software, Boston, MA, USA). Parametric data were analysed using Student’s *t*-test or ANOVA and Tukey post-test. Non-parametric data were analysed using Mann–Whitney test or Kruskal–Wallis test with Dunn’s Multiple Comparison test as post-test. Statistical significance was expressed with minimum *p* < 0.05 (*), *p* < 0.01 (**) and *p* < 0.001 (***).

## 3. Results

### 3.1. Formulations and Practical Use

**Figure 3:**
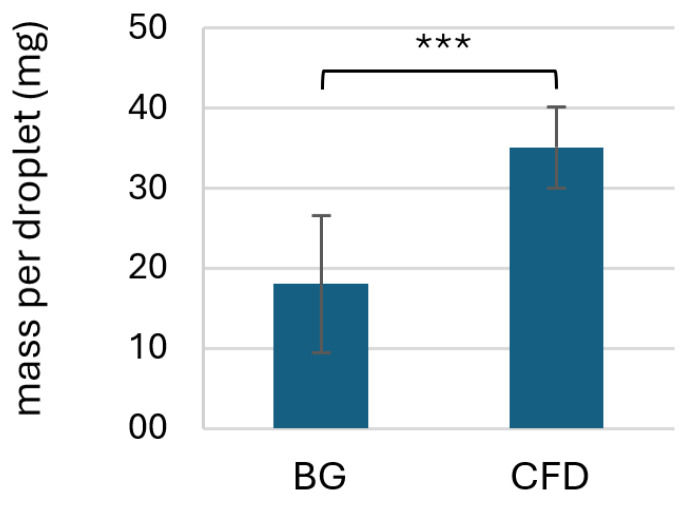
Comparison of dispensed droplet volume of AS eye drops (0.01% *w*/*w*) using two different dispenser types (BG = brown glass with BIIR cone; CFD = contamination-free LDPE dispenser unit of the Novelia^®^ type). Values represent means of *n* = 80 ± SD dispensed units (acquired on different days by the same experimenter in a total of *n* = 8 experiments with 10 technical replicates each). Statistically significant differences are marked with asterisks (*** *p* < 0.001, Mann–Whitney test).

Preliminary experiments were conducted to evaluate the dispensed volume and estimate the required amount of residual volume for microbial and chemical stability testing for both the CFD and BG (with BIIR dripping cone) dispenser bottles. Results showed that BG-type dispensers led to significantly lower droplet mass per application if only shaking was employed to dispense the product, while CFD units led to a higher and more consistent dispensed droplet volume (Figure 3).

Since squeezing of the dripping cone to achieve a higher droplet volume was deemed less hygienic, dripping without manual manipulation of the BG dispensing unit was used for all further studies. An additional stress test (tip seal integrity test) was, however, included in the experimental setup.

### 3.2. Microbial Stability via Agar Plating

**Figure 4:**
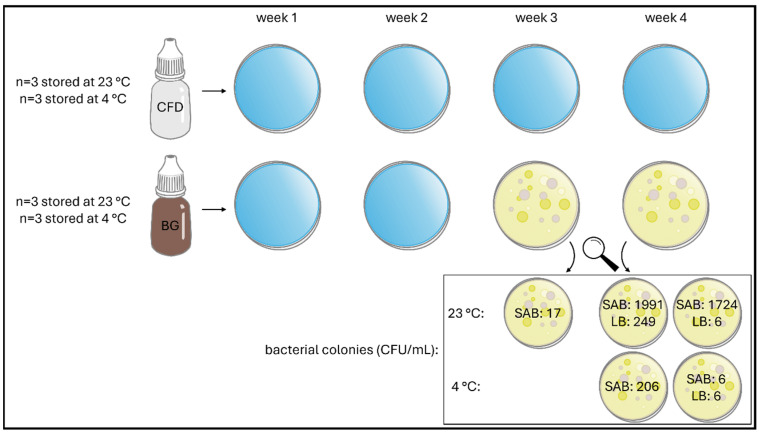
Illustration of contamination record of unpreserved AS eye drops (0.01% *w*/*w*) during agar plating over 4 weeks of daily dripping simulation. Two dispenser types (BG = brown glass; CFD = contamination-free LDPE Novelia^®^ dispenser) and two storage temperatures (23 °C, 4 °C) were tested. Individual bacterial colonies per contaminated sample are shown for SAB and LB agar media.

**Figure 5:**
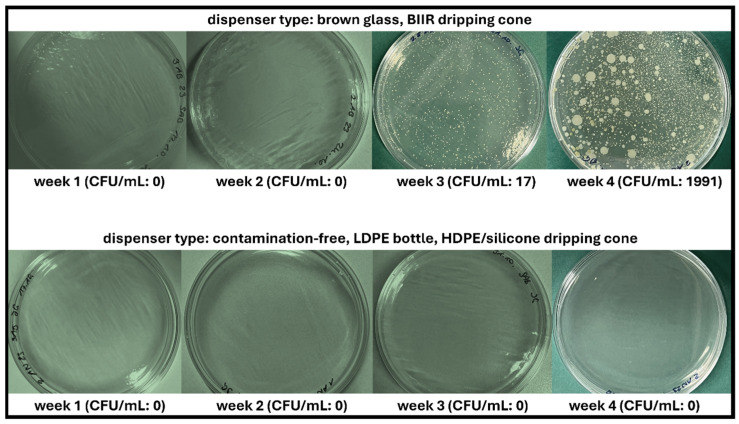
Colony-forming units (CFU) of bacteria observed during agar plating of unpreserved AS eye drops (0.01% *w*/*w*) in different dispenser types during daily use over 4 weeks. Representative images are shown (storage temperature 23 °C, agar media SAB).

Since the first few weeks of daily use of eye drops after aseptic production are particularly important and served to compare CFD advanced dispensers for unpreserved eye drops and classic multidose (BG) dispensers, plating was conducted every 7 days after opening and daily use for samples stored at 23 °C and 4 °C, and visual contamination was documented (Table 1 and Figure 4 and Figure 5).

No contamination with bacteria or fungi was observed in either agar plate/medium after week 1 and week 2, regardless of the dispenser type and storage temperature. In week 3, bacterial contamination was detected for BG samples stored at 23 °C, and in week 4, bacterial contamination was also detected for BG samples stored at 4 °C. No contamination with fungi was observed in either agar medium. In summary, all BG dispenser samples were deemed unsafe regardless of storage temperature since patient adherence regarding hygienic use and adequate storage cannot be ascertained under real-life conditions. The requirements of the *European Pharmacopoeia* were therefore confirmed, limiting the use of unpreserved aqueous eye drops in conventional multidose dispensers to 24 h after opening. In contrast, Novelia^®^ samples were not affected by contamination introduced during daily dripping and remained contamination-free at both storage temperatures (23 °C and 4 °C).

### 3.3. Microbial Stability via Direct Inoculation (Ph.Eur. 2.6.1.)

**Figure 6:**
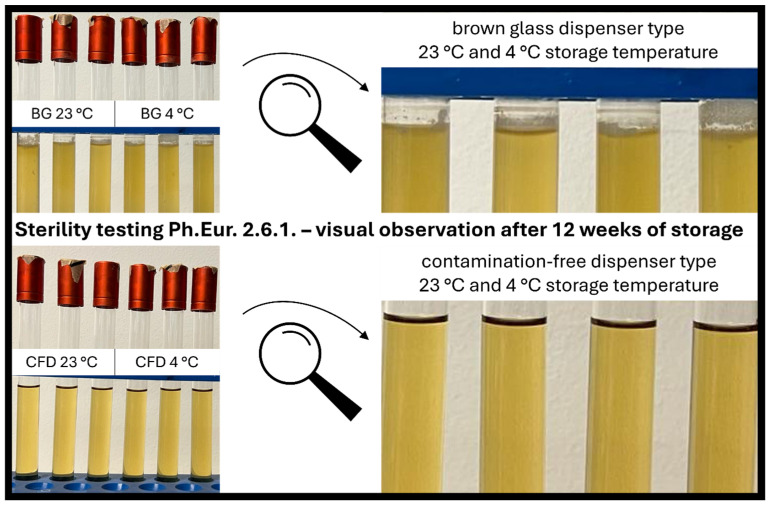
Visual assessment of product sterility of AS eye drops (0.01% *w*/*w*) after 12 weeks of in-use simulation. Two dispenser types (BG = brown glass; CFD = contamination-free LDPE Novelia^®^ dispenser) and two storage temperatures (23 °C, 4 °C) were tested. Representative images of direct inoculation experiments (Ph.Eur. 2.6.1. after 14 days of incubation) are shown to visualise contamination processes.

Sterility assessment according to the *European Pharmacopoeia* confirmed the findings of the plating experiments after week 4 of in-use simulation and gave additional information on microbial stability after week 8 and week 12 of eye drop use. For all samples (week 4, week 8, week 12), eye drops in CFD showed no signs of contamination and can thus be considered safe to use both when stored at 23 °C and 4 °C (Figure 6).

In contrast, all samples stored in conventional BG dispensers showed contamination at all time points: week 4 samples showed contamination after 3 days of incubation, and week 8 and week 12 samples showed 100% contamination after only 1 day of incubation. As expected, there was a slight tendency towards higher contamination rates for samples stored at 23 °C vs. 4 °C, which does not affect the general outcome that the conventional BG dispenser type is unsafe to use for unpreserved eye drops, while the CFD type appears to be safe to use over 12 weeks.

### 3.4. Tip Seal Integrity Test (TSIT)

**Figure 7:**
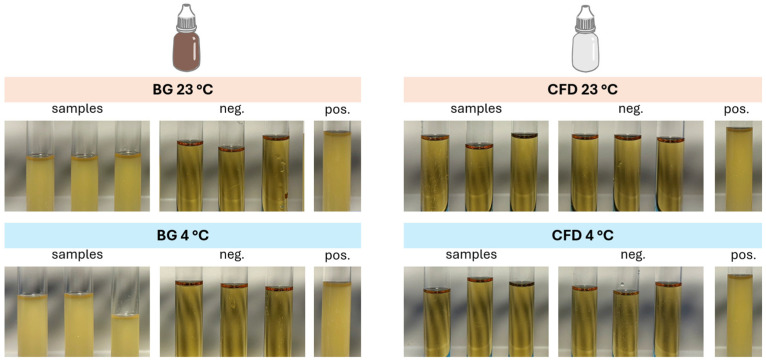
Optical appearance of nutrition media removed from the different dispenser types (BG = brown glass; CFD = contamination-free LDPE Novelia^®^ dispenser) after the tip seal integrity test (negative control = sterilised CASO-B medium, positive control = sterilised CASO-B medium + *E. coli* DH5α). Experiments were conducted in triplicate (*n* = 3).

To additionally check the risk of microbial contamination during use, a microbial tip seal challenge test was conducted. In line with the negative controls, samples stored in CFD of the Novelia^®^ type remained free from contamination both after storage at 23 °C and 4 °C (Figure 7). In contrast, BG dispenser samples showed strong contamination in all cases after the challenge, regardless of storage temperature.

These findings confirm the suitability of the CFD for the application of compounded eye drop solutions.

### 3.5. HPLC Assessment of Drug Content

**Figure 8:**
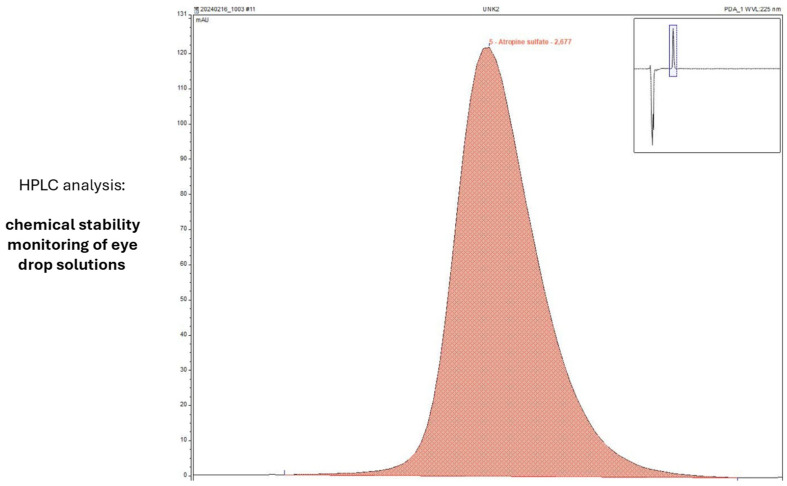
Representative quality control chromatogram during the in-use simulation study of AS eye drops (0.01% *w*/*w*).

**Figure 9:**
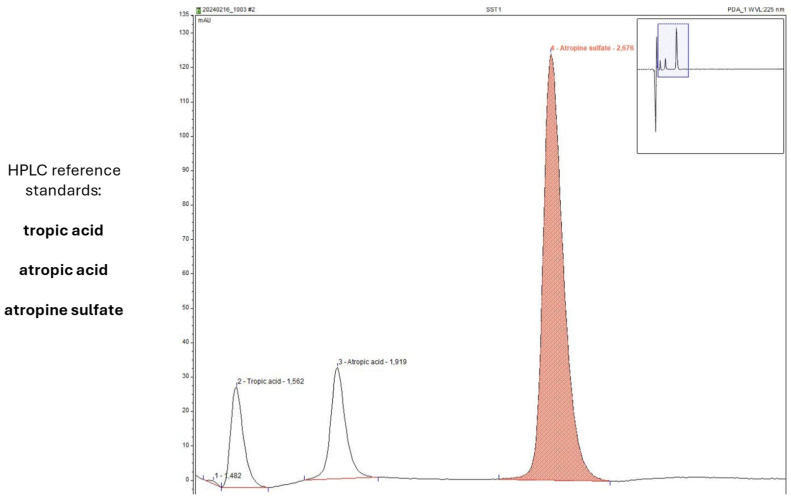
Representative chromatogram of sample analysis during the in-use simulation study of AS eye drops (0.01% *w*/*w*).

Information on chemical stability and drug content was collected using the same in-use conditions (two dispenser types, storage at 23 °C and 4 °C). HPLC analysis was used to determine AS content (Figure 8) and scan for known degradation products (Figure 9). Drug content could be determined with high accuracy, and no degradation products were detected after 4, 8 or 12 weeks of storage.

**Figure 10:**
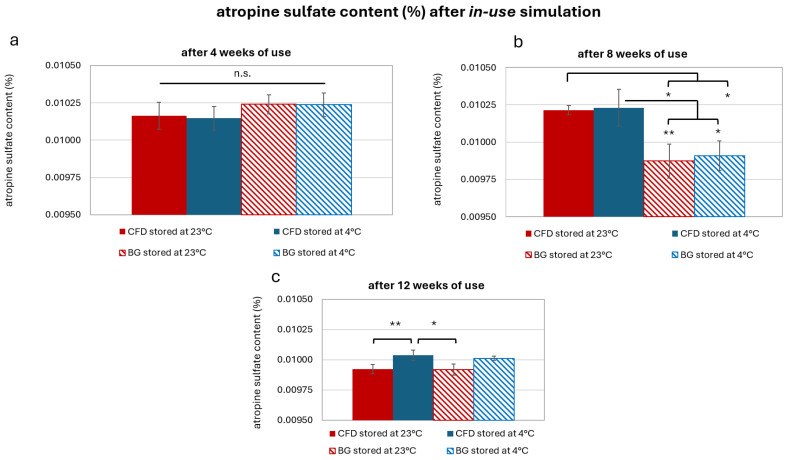
Atropine sulfate (AS) content after (**a**) 4, (**b**) 8 and (**c**) 12 weeks of storage in different dispenser types (CFD = contamination-free LDPE dispenser of Novelia^®^ type; BG = brown glass with BIIR cone) at either 23 °C or 4 °C storage. Results are means ± SD of *n* = 3. Statistically significant differences in AS content are marked with asterisks (* *p* < 0.05, ** *p* < 0.01, n.s. = no significant differences, paired Student’s *t*-test).

Results showed that drug content remained in the envisioned concentration range of 0.01% *w*/*w* AS in weeks 4, 8 and 12 (Figure 10). In week 4, drug content was identical regardless of dispenser type and storage temperature (*p* < 0.05, Figure 10a). In week 8, lower drug content was observed for samples stored at 23 °C and in BG dispensers (*p* < 0.05 or 0.01, Figure 10b). However, this could have been caused by methodological fluctuations in the HPLC analysis, as the differences between dispenser types were not observed at week 12 (*p* > 0.05, Figure 10c). Week 12 samples stored at 4 °C did show significantly higher AS content than samples stored at 23 °C (*p* < 0.05 or 0.01). Therefore, refrigerated storage should be recommended in daily practice to optimise therapeutic success.

In summary, chemical stability monitoring via HPLC showed that the envisioned AS content of 0.01% *w*/*w* remained within a plausible range after 12 weeks of regular use (± 0.0002%, no degradation products detectable). Considering references in the literature working with thresholds of 90–110% of initial drug content as acceptable for therapeutic approaches using compounded products [29], AS content remained in a highly satisfying range.

### 3.6. Osmolality and pH Assessment

**Figure 11:**
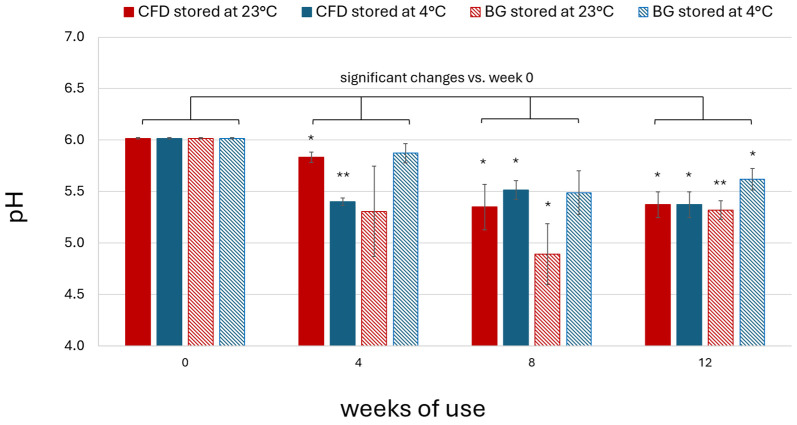
pH of unpreserved AS eye drops (0.01% *w*/*w*) during in-use simulation of 4, 8 and 12 weeks using different dispenser types (CFD = contamination-free LDPE dispensers of Novelia^®^ type; BG = brown glass dispenser with BIIR cone) and storage conditions (23 °C vs. 4 °C). Results are means ± SD of *n* = 3 samples per batch. Statistically significant differences in AS content are marked with asterisks (* *p* < 0.05, ** *p* < 0.01, paired Student’s *t*-test).

Osmolality of AS (0.01% *w*/*w*) eye drop solutions was found to be around 285 mOsm/kg (284.67 ± 2.05). For characterisation and stability monitoring, pH was assessed during production and after 4, 8 and 12 weeks of in-use simulation. The initial formulation pH value was around pH 6. A decrease in pH was observed after opening and daily dripping simulation for all dispenser types and at both storage temperatures (Figure 11). The general pH decrease did not always follow a consistent pattern in relation to storage time, dispenser type and temperature. However, the lowest pH was always observed in BG dispensers stored at 23 °C, which can therefore be considered to provide the lowest protection against pH change caused by environmental exposure. Summarised on average for week 4, 8 and 12 samples stored in CFD units, pH decreased by roughly 8% at 23 °C (8.27 ± 3.70%) and 10% at 4 °C (9.75 ± 1.03%). In BG dispensers, pH decreased by roughly 14% at 23 °C (14.02 ± 3.29%) and 6% at 4 °C (5.91 ± 2.65%). In the case of the BG dispenser type, storage temperature appeared to play an important role in pH stability, which correlated with the higher contamination observed in these samples.

In summary, a decrease in pH was observed to be acceptable for AS eye drops (0.01% *w*/*w*) in CFD stored at 23 and 4 °C and BG dispensers at 4 °C, but not for BG dispensers stored at 23 °C.

It is well established that the pH of ophthalmological products should ideally match the physiological pH of tear fluid (7.4–7.6) or be within the ocular comfort range (6.6–7.8) to avoid lacrimation, pain and discomfort upon application [33], which is especially important for adherence of young patients. The observed pH range of unpreserved AS (0.01% *w*/*w*) eye drops was around pH 6, with a decrease by roughly 10% to values around 5.4 in CFD units after four weeks of use. This indicates that use of a buffer system might be advisable for increased patient comfort; however, many marketed eye drop products exhibit acidic pH to avoid chemical disintegration of incorporated drugs (e.g., pilocarpine hydrochloride exhibits optimal chemical stability at pH 4 [34]). The observed pH of the investigated compounded product is clinically acceptable, although the pH decrease during storage may reduce patient comfort over time. Evaluation of different buffered drug solutions would be required to improve patient comfort; however, this may potentially increase the risk of AS hydrolysis [35].

## 4. Discussion

The data presented here constitute a pilot study aimed at establishing stability data for the safe use of compounded eye drops in Austrian pharmacy practice. Not within the scope of this pilot project was the evaluation of different drug concentrations used in daily practice (0.01–2% *w*/*w* AS) with respect to microbial contamination and chemical stability. A previous study by Saito et al. [13] found microbial stability of unopened AS eye drop solutions in different concentrations (0.1, 1.0, 2.5, 5.0 mg/mL) in multidose polyethylene eyedropper bottles stored at 25 °C and 5 °C for 6 months, as well as satisfying drug content (±5% *w*/*w* of the target value), which is in good agreement with our data on AS content after 3 months of use.

Another important topic is the pH of compounded AS eye drop solutions, as it may affect the chemical stability of the drug as well as its efficacy at the site of action through modified penetration potential [9]. It is well known that AS is prone to degradation in aqueous solutions of higher pH, and therefore, the pH of compounded AS eye drops should be defined and monitored to ensure consistent clinical results despite local variations regarding eye drop composition (e.g., use of preserving agents vs. unpreserved eye drops) [36]. For unpreserved AS eye drops, Saito et al. reported a pH decrease in unopened dispensers at all tested AS concentrations [13]; for AS content of 0.01% *w*/*w*, pH decrease amounted to approximately 4% after 12 weeks at both 25 °C and 5 °C storage temperatures. This trend was confirmed by our pH data, although the pH drop observed during our in-use simulation was more pronounced, with a 10% pH decrease in CFD after opening at both 23 °C and 4 °C storage. This may be associated with the increased ventilation of the dispenser systems caused by depletion of the eye drop solutions during use.

**Figure A1:**
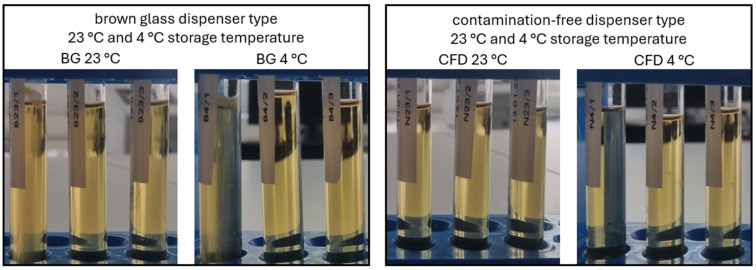
Visual assessment of product sterility of HHBr eye drops (1% *w*/*w*) after 12 weeks of in-use simulation. Two dispenser types (BG = brown glass; CFD = contamination-free LDPE Novelia^®^ dispenser) and two storage temperatures (23 °C, 4 °C) were tested (direct inoculation Ph.Eur. 2.6.1. after 14 days of incubation).

**Figure A2:**
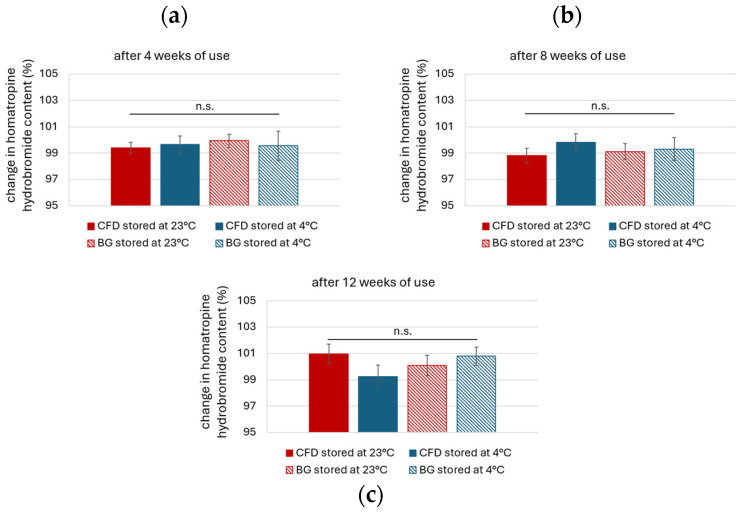
Homatropine hydrobromide (HHBr) content after (**a**) 4, (**b**) 8 and (**c**) 12 weeks of storage in different dispenser types (CFD = contamination-free LDPE dispenser; BG = brown glass with BIIR cone) at either 23 °C or 4 °C storage. Results are means ± SD of *n* = 3 (n.s. = no significant differences).

**Figure A3:**
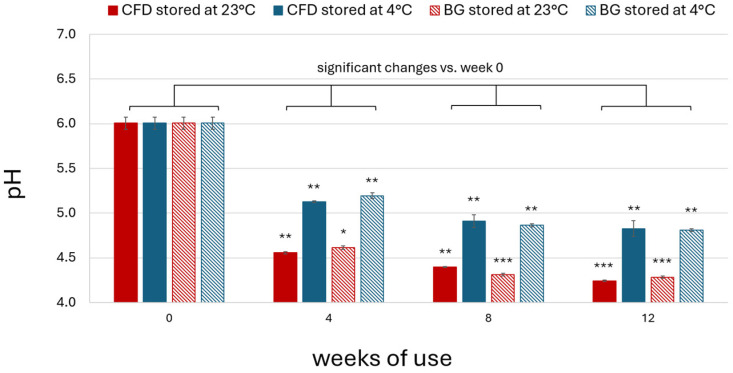
pH of unpreserved HHBr eye drops (1% *w*/*w*) during in-use simulation of 4, 8 and 12 weeks using different dispenser types (CFD = contamination-free LDPE dispensers of Novelia^®^ type; BG = brown glass dispenser with BIIR cone) and storage conditions (23 °C vs. 4 °C). Results are means ± SD of *n* = 3 samples per batch. Statistically significant differences in AS content are marked with asterisks (* *p* < 0.05, ** *p* < 0.01, *** *p* < 0.001, paired Student’s *t*-test).

However, we would like to point out the importance of pH assessment in addition to monitoring microbial stability and sufficient drug content via HPLC during in-use simulation. Additional studies were performed on a different classic compounded eye drop product, homatropine hydrobromide (1% *w*/*w*) eye drops for diagnostic purposes (see Appendix A). Results confirmed microbial safety (Figure A1) and stable drug content (Figure A2 and Figure A3) for CFD units over 4, 8 and 12 weeks of dripping simulation, confirming the results of the main studies on AS eye drops. However, a drop in pH of 27% at 23 °C and 18% at 4 °C was observed for homatropine hydrobromide eye drops, regardless of dispenser type (Figure A3), suggesting a strong need for cooled storage and/or use of buffering systems to avoid ocular irritation due to the acidic pH.

For aseptic production of the eye drop solutions, open filtration in an LAF was chosen as a standard method established in Austrian compounding practice. No setup was implemented for comparison with different aseptic production methods, such as direct filtration within the sterile packing unit outside of an LAF, as is common practice in German compounding routines. If performed correctly, both methods lead to safe ophthalmological products. A recent report by Wongwirawat et al. [11] describes the production of AS (0.01% *w*/*w*) eye drops from commercial solutions for injection. Although the methodology involves compounding using 15 mL eye drop dispensers, which are not acceptable according to European regulatory standards, and sterilisation using ethylene oxide, the daily dripping simulation suggested acceptable AS content and no microbial contamination over 12 weeks of use, which agrees with our findings.

In-use safety of the unpreserved AS eye drops was analysed over 12 weeks after opening, which is important for establishing a consistent and safe treatment regime for young patients. So far, in-use safety over 4 weeks has been suggested for the tested CFD unit (Novelia^®^) in studies conducted in French hospital pharmacy practice [37]. In our studies, no prolonged use after week 12 was investigated. The data discussed are therefore only valid for the given simulated use (two droplets/day, 5 workdays/week), which led to product depletion after approximately 12 weeks. For products that are not used daily (intermittent treatment regime [6]) and that could therefore suffice for longer time spans than 12 weeks, separate studies would be required to evaluate in-use stability. In addition, no systematic microbial analysis of the next dose dispensed from CFD units after the contamination stress test was conducted, but it will be subject to follow-up studies. Preliminary tests suggested risk for contamination and should be extended to establish sanitary protocols, e.g., disinfection of the dispenser tip, to exclude external contamination risks.

## 5. Conclusions

Unpreserved AS eye drops (0.01% *w*/*w*), as regularly produced in pharmaceutical compounding to treat pediatric myopia, can be produced under aseptic conditions using multidose CFD units. If state-of-the-art production, storage conditions and correct use are maintained, the observed in-use shelf life of 12 weeks regarding microbial stability and drug content is an important asset to facilitate efficient long-term treatment of patients and avoid excessive plastic waste associated with using mono-dose dispensing units. A decrease in pH over time could be counteracted by the use of an appropriate buffer system in the future.

## Figures and Tables

**Table 1:** Contamination record of unpreserved AS eye drops (0.01% *w*/*w*) during agar plating over 4 weeks of daily dripping simulation. Two dispenser types (BG = brown glass; CFD = contamination-free LDPE Novelia^®^ dispenser) and two storage temperatures (23 °C, 4 °C) were tested. Results of three individual batches (labels 1, 2, 3) are shown. Contamination was labelled as free of colony-forming units (-) or contaminated (+, ++ and +++ for low, intermediate and high contamination).

Sample Batch	Week 1	Week 2	Week 3	Week 4
1-CFD 23	-	-	-	-
1-CFD 4	-	-	-	-
1-BG 23	-	-	-	-
1-BG 4	-	-	-	++
2-CFD 23	-	-	-	-
2-CFD 4	-	-	-	-
2-BG 23	-	-	-	+++
2-BG 4	-	-	-	-
3-CFD 23	-	-	-	-
3-CFD 4	-	-	-	-
3-BG 23	-	-	+	+++
3-BG 4	-	-	-	+

## Data Availability

The dataset is available upon request from the authors.

## Abbreviations

The following abbreviations are used in this manuscript:

AS	atropine sulfate
BG	brown glass
BIIR	bromobutyl rubber
CASO	casein peptone agar
CASO-B	casein peptone bouillon
CFD	contamination-free dispenser
CFU	colony-forming unit
*E. coli*	*Escherichia coli*
HDPE	high-density polyethylene
HHBr	homatropine hydrobromide
LAF	laminar air flow
LB	lysogeny broth medium (Lennox)
LDPE	low-density polyethylene
PET	polyethylene terephthalate
SAB	Sabouraud 4% dextrose agar
TSIT	tip seal integrity test

## Appendix A. Homatropine Hydrobromide 1% *w*/*w* Eye Drops

### Appendix A.1. Production and Analysis of Homatropine Hydrobromide 1% w/w Eye Drops

Isotonic eye drops were produced by dissolving 0.4 g homatropine hydrobromide (HHBr, Ph.Eur., CAS-Nr. 51-56-9, Fagron GmbH, Glinde, Germany) in 32.95 g isotonic pyrogen-free saline mixed with 6.65 g aqua ad injectabilia to obtain a final concentration of 1% *w*/*w* (i.e., 10 mg/mL), followed by sterile filtration and aseptic filling of 10 mL eye drop solution into sterile LDPE bottles or sterile brown glass bottles. Four eye drop units per batch (two CFD, two BG dispensers) were produced.

Storage and simulated patient use were conducted as described in the main text. Microbial stability was monitored by direct inoculation, and physico-chemical stability was assessed by HPLC and pH monitoring after 4, 8 and 12 weeks on separate batches as described in the main text.

For HPLC analysis, an adapted protocol following the directives of the USP monograph *Homatropine Hydrobromide* was used. Samples were analysed using a Shimadzu HPLC instrument with diode array detection (2 × SHIMADZU degasing unit DGU-20A5R, 1 × SHIMADZU degasing unit DGU-20A3R, SHIMADZU LC-30AD pump A, SHIMADZU LC-30AD pump B, SHIMADZU communication bus module, SHIMADZU SIL-30AC autosampler, SHIMADZU RF-20A XS fluorescence detector, SHIMADZU SPD-M20A diode array detector, SHIMADZU CTO-30A column oven, Shimadzu Ges.m.b.H., Korneuburg, Austria). An octadecyl silane-based column in reversed-phase mode (LUNA C18(2) 100mm x 4.6mm x 3 µm, Phenomenex Ltd., Torrance, CA, USA) was employed as the stationary phase. The mobile phase consisted of methanol/ potassium phosphate buffer (pH 2.7 (33:67 *v*/*v*; buffer composition: 6.8 g monobasic potassium phosphate and 7.0 g sodium 1-heptanesulfonate monohydrate per 1 L of distilled water)). After degassing, samples were injected at a flow rate of 1.5 mL/min at an oven temperature of 40 °C; the injection volume was set at 7 µL. Chromatograms were analysed at a PDA range of 200–400 nm. Peak areas (AUC) were obtained using the Chromeleon 7 Chromatography Data System (Thermo Scientific Chromeleon Version 7.2.10 ES). Samples were analysed for HHBr content and the content of known impurities like mandelic acid, dehydrohomatropine, scopolamine and atropine. Drug quantification was performed using reference solutions within a calibration range of 0.5–2.5 mg/mL and an R^2^ of 1.000.

### Appendix A.2. Stability Data on Homatropine Hydrobromide 1% w/w Eye Drops

Sterility assessment for HHBr eye drops showed similar results as observed in the main study: eye drops in CFDs revealed no signs of contamination after 4, 8 or 12 weeks (Figure A1) and can thus be considered safe for use irrespective of storage temperature (23 or 4 °C), as opposed to BG dispensers (contamination).

HHBr content after 4, 8 and 12 weeks of simulated use and scanning for known degradation products was performed via HPLC. Drug content remained in the range of 1% *w*/*w* after 4, 8 and 12 weeks (Figure A2, 98.8–101.0% vs. week 0). Neither dispenser type nor storage temperature affected the outcome, which indicates lower dependency on package material and temperature than observed for AS.

The pH of HHBr (1% *w*/*w*) eye drops was measured during production and after 4, 8 and 12 weeks of simulated use. Initial pH was around 6, like for the AS product. As for AS eye drops, results showed a decrease in pH after daily dripping for all dispenser types and storage temperatures (Figure A3), which was comparable for CFD and BG dispensers and stronger at 23 °C. Averaging all samples for the two dispenser types, pH decreased by 27% at 23 °C (CFD: 26.77 ± 2.61%; BG: 26.69 ± 3.04%) and by 18% at 4 °C (CFD: 17.52 ± 2.58%; BG: 17.50 ± 3.46%).

In conclusion, albeit stable regarding microbial aspects and drug content, unpreserved HHBr eye drops (1% *w*/*w*) showed a decrease in pH > 10% of the initial value for both dispenser types and storage temperatures after 4, 8 and 12 weeks.

As reported for AS (0.01% *w*/*w*) eye drops, compounded HHBr eye drops can be assumed to be safe and effective over 12 weeks of storage in CFD units if production, use and storage conditions are adequate. However, the significant drop in pH after 4 weeks indicates the need for buffering systems to ensure physiologically acceptable pH.
